# Clade 2.3.4.4b highly pathogenic avian influenza H5N1 viruses: knowns, unknowns, and challenges

**DOI:** 10.1128/jvi.00424-25

**Published:** 2025-05-09

**Authors:** Zimin Xie, Jiayun Yang, Wanlin Jiao, Xueqing Li, Munir Iqbal, Ming Liao, Manman Dai

**Affiliations:** 1 National and Regional Joint Engineering Laboratory for Medicament of Zoonosis Prevention and Control, Guangdong Provincial Key Laboratory of Zoonosis Prevention and Control, College of Veterinary Medicine, South China Agricultural University 12526, Guangzhou, China; 2 UK-China Center of Excellence for Research on Avian Diseases, Guangzhou, China; 3 The Pirbright Institute, Pirbright, Woking, United Kingdom; Indiana University Bloomington, Bloomington, Indiana, USA

**Keywords:** highly pathogenic avian influenza, H5N1, evolution, pathogenicity, Clade 2.3.4.4b

## Abstract

Since 2020, the clade 2.3.4.4b highly pathogenic avian influenza (HPAI) H5N1 viruses have caused unprecedented outbreaks in wild birds and domestic poultry globally, resulting in significant ecological damage and economic losses due to the disease and enforced stamp-out control. In addition to the avian hosts, the H5N1 viruses have expanded their host range to infect many mammalian species, potentially increasing the zoonotic risk. Here, we review the current knowns and unknowns of clade 2.3.4.4b HPAI H5N1 viruses, and we highlight common challenges in prevention. By integrating our knowledge of viral evolution and ecology, we aim to identify discrepancies and knowledge gaps for a more comprehensive understanding of the virus. Ultimately, this review will serve as a theoretical foundation for researchers involved in related avian influenza virus studies, aiding in improved control and prevention of H5N1 viruses.

## INTRODUCTION

Influenza A viruses (IAVs) are important zoonotic pathogens, posing a credible concern to animals and humans. The viral genome is negative-sense, single-stranded, and consists of eight segments. IAVs are classified into 19 hemagglutinin (HA) subtypes and 11 neuraminidase (NA) subtypes (1). H17N10 and H18N11 have only been identified in bats, while H1–H16, H19, and N1–N9 can infect a wide range of avian and mammalian hosts, including humans (2, 3). Wild birds are the natural reservoirs for IAV, where they are referred to as avian influenza viruses (AIVs). All 16 HA subtypes and 9 NA subtypes have been identified in wild birds (4).

Since 2020, many countries have reported outbreaks of clade 2.3.4.4b highly pathogenic avian influenza (HPAI) H5N1 viruses, leading to devastating economic losses (5–9). Domestic birds, like chickens, typically experience a 100% mortality rate following infection with H5 HPAIVs. Wild birds, on the contrary, are naturally resistant to HPAIVs, exhibiting no or mild clinical symptoms upon HPAIV infection (10, 11). However, the contemporary clade 2.3.4.4b H5N1 viruses have caused unprecedented infections in wild bird populations with high mortality rates (5, 12–14). In addition to avian species, many countries have reported the infections of clade 2.3.4.4b H5N1 virus in mammalian species including foxes, domestic cats, minks, sea lions, and most recently in dairy cows (7, 9, 13, 15–17). Moreover, sporadic zoonotic infections have been reported in several countries (18–20). Altogether, this suggests that the current clade 2.3.4.4b HPAI H5N1 viruses pose significant threats to human and animal health. The review delves into published data of clade 2.3.4.4b H5N1 HPAIVs. We will also shed light on the current situation of the virus and highlight potential avenues for future research.

## EMERGENCE, TRANSMISSION, AND EVOLUTION OF CLADE 2.3.4.4b HPAI H5N1 VIRUSES

**Fig 1:**
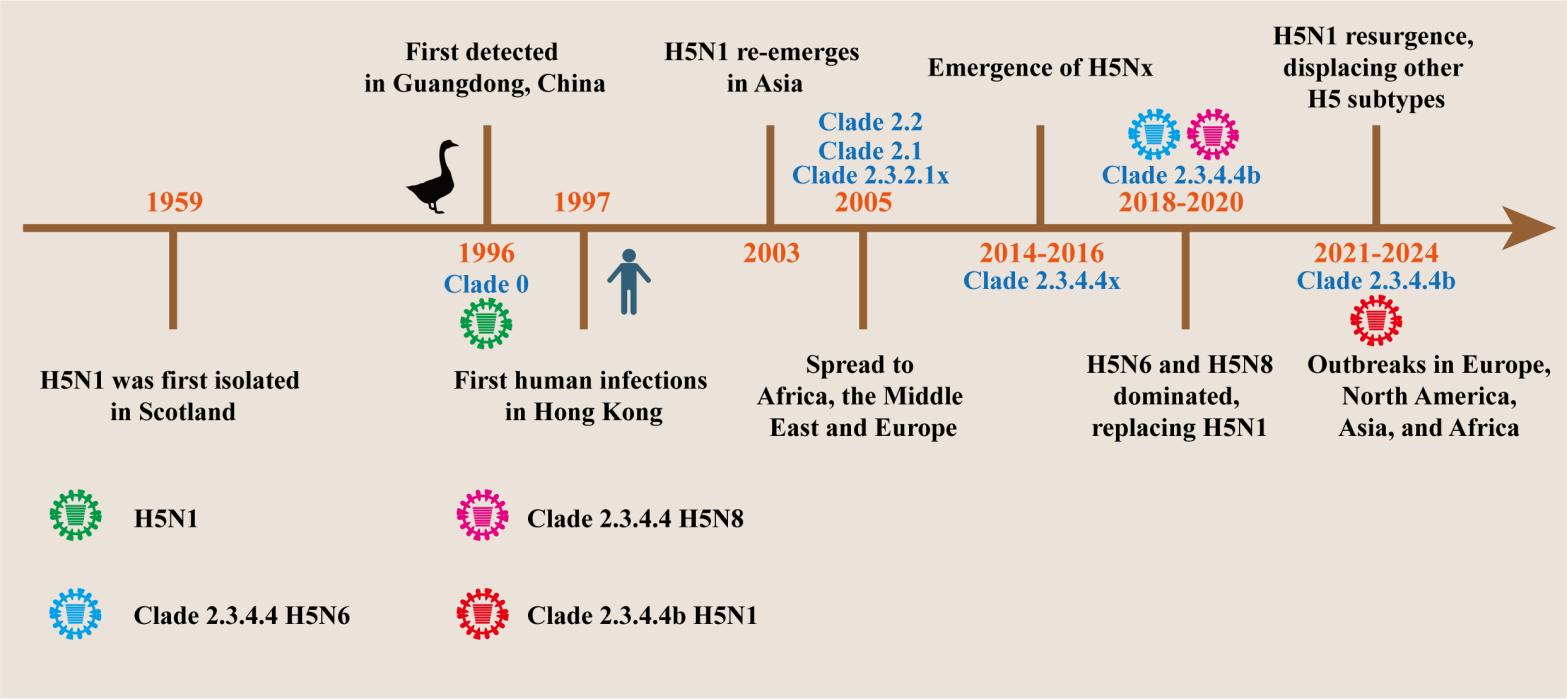
Timeline of HPAI H5N1 evolution. This timeline shows significant events and important clades of HPAI H5N1.

HPAIVs are distinguished from low pathogenicity H5 AIVs by the presence of multi-basic cleavage site in the HA. Since 1996, the HPAI A/goose/Guangdong/1/96 (Gs/GD/96) lineage viruses have been prominent, although other lineages have also circulated, such as the H5N2 HPAIV strains that emerged in Mexico in the mid-1990s (Fig. 1) (21). The Gs/GD/96 lineage demonstrated zoonotic potential, with 18 reported human infections and six fatalities in Hong Kong in 1997 (22–24). This marked the first documented instance of H5N1 HPAIV transmission from birds to humans. Following the closure of live poultry markets by local authorities, the H5N1 virus stayed undetected in domestic birds until 2002, when outbreaks in poultry farms resulted in human fatalities (25, 26). Three years later, the H5N1 virus was also detected in migratory birds in Qinghai Lake, China (27, 28). Since then, HPAI H5 viruses remain persistent and continue to spread among bird populations worldwide (29). Currently, the Gs/GD/96 HPAIV lineage H5 subtype has rapidly evolved into over 30 clades and subclades resulting in substantial antigenic diversity (30). Between 2005 and 2014, clades 2.1, 2.2, and 2.3.1.2a-c remained persistent compared to other clades (27, 31–33). Since 2014, clade 2.3.4.4 emerged and became dominant in Asia, Europe, and North America with multiple genotypes such as H5N2, H5N6, and H5N8, collectively termed as H5Nx, where Nx is N1–N9 (34–37). By the end of 2015, H5N1 of previous clades had been replaced by clade 2.3.4.4 H5Nx viruses (38). Clade 2.3.4.4 has been meticulously analyzed and categorized into eight subclades, 2.3.4.4a to h, and 2.3.4.4b is the most dominant subclade (39, 40). Clade 2.3.4.4b H5N8 remained prevalent in wild birds until reassortment with other avian influenza strains led to the emergence of a novel H5N1 in 2020 (41).

Clade 2.3.4.4b H5N1 viruses spread rapidly across Eurasia and Africa by fall 2021, becoming the dominant subtype globally (42). Bird migration likely facilitated its arrival in North America by early 2022 (16, 36). The virus has now been widely detected in Europe, Southeast Asia, and the Antarctic region in autumn 2023 (9, 15, 16, 43–45). To date, more than 100 reassortant H5N1 genotypes have been identified in North America, with over 50 genotypes documented in Europe, demonstrating substantial diversity within clade 2.3.4.4b HPAI H5N1 viruses (5, 39). The rapid evolution and reassortment of the H5N1 virus underscore the urgent need to monitor and assess the risk of emerging variants to mitigate future risks.

**Fig 2:**
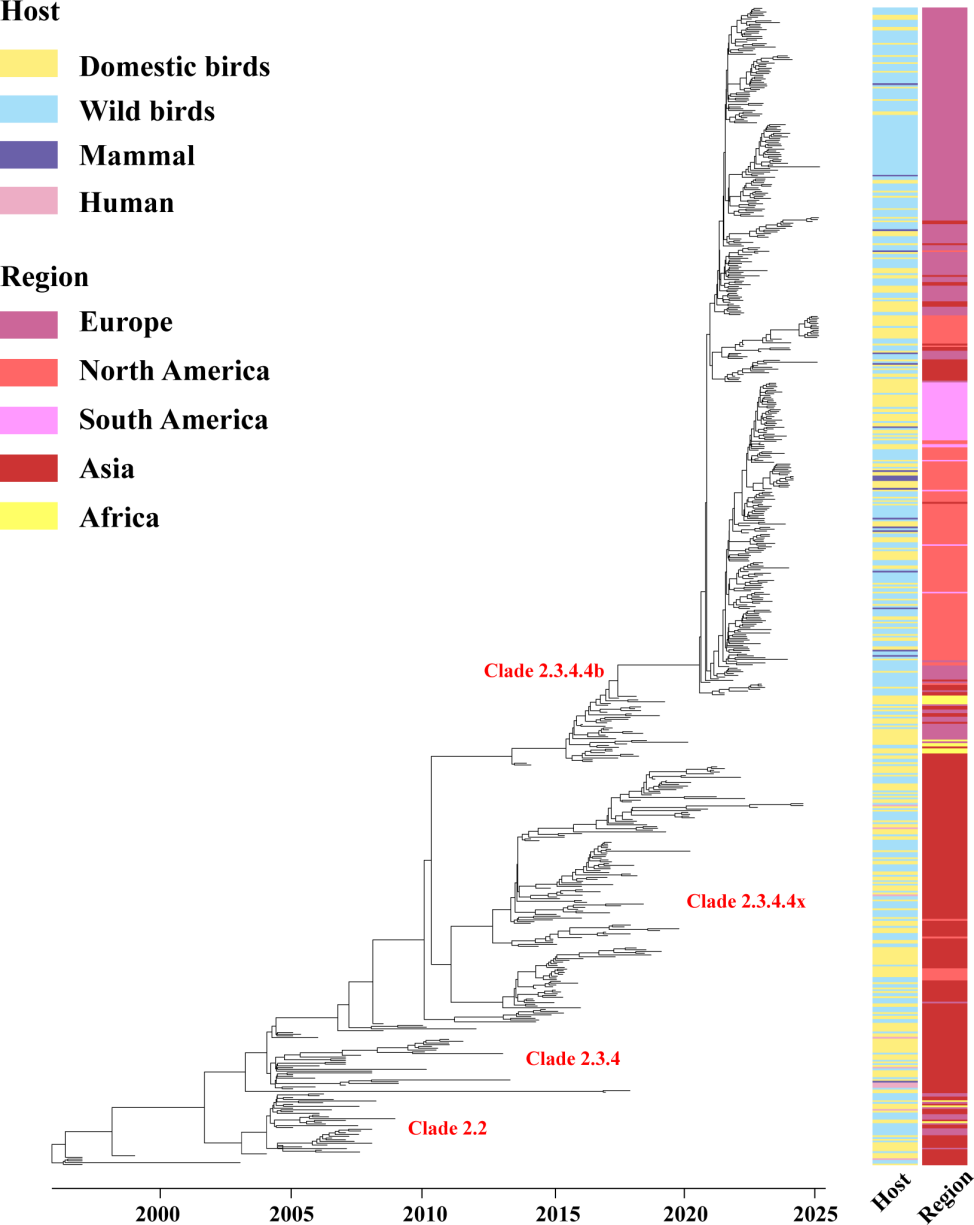
Genetic evolution of HPAI H5N1 HA gene from 1997 to 2023. All available H5 subtype AIV HA gene sequences were retrieved from GISAID (https://www.gisaid.org/). Multiple sequence alignment was conducted using the MAFFT program. All sequences were subjected to data cleaning using Cluster Database at High Identity with Tolerance (CD-HIT) version 4.8.1, where sequences were removed if they met the following criteria: (i) containing multiple ambiguous bases and (ii) having a similarity of 99.5% or higher. The best-fit model under the Bayesian framework was determined using ModelFinder. We utilized BEAST v.1.10.4 to run the phylogeny tree, with the best-fit model being GTR + F + I + G4. Divergence times and evolutionary rates were estimated using an uncorrelated relaxed clock model and Bayesian Skygrid model. Bayesian Markov chain Monte Carlo was set to 200 million total iterations, with sampling every 20,000 iterations.

AIV glycoprotein HA plays a crucial role in modulating antigenicity. To gain a comprehensive understanding of the current evolutionary trend of clade 2.3.4.4b HPAI H5N1, HA sequences of H5 subtype viruses since 1997–2024 were acquired from the Global Initiative on Sharing All Influenza Data (GISAID). From GISAID, we obtained approximately 10,000 HA sequences from the H5 subtype. For this analysis, we refined the data set to 782 sequences based on criteria prioritizing geographic distribution, collection date, clade, and host type to maintain a representative and manageable sample size for maximum likelihood analysis (Fig. 2; Data S1) (46, 47). The Bayesian phylogenetic tree of the HA gene indicates that clade 2.3.4.4b HPAI H5N1 has been detected in Europe, North America, Asia, and South America. Lineages in North America and South America are similar and directly originated from the Eurasian lineage. Notably, the strains in Asia form a distinct clade closely related to the European strains. This suggests that Asia may serve as the primary origin of the 2.3.4.4b H5N1 lineage. Moreover, similarity analysis of the HA gene revealed that the nucleotide sequence homology ranges from 96.3% to 99.5%, and the amino acid sequence homology ranges from 95.1% to 100%. These findings collectively suggest that HA is relatively conserved despite the rapid evolution of clade 2.3.4.4b HPAI H5N1. Based on the fact that the clade 2.3.4.4b HPAI H5N1 viruses have caused unprecedented infections in broader geographic locations and more expanded host range, it is likely that the reassortment and adaptation of internal genes and NA likely contribute to the virus fitness (13, 35).

## EXPANDED HOST RANGE OF CLADE 2.3.4.4b HPAI H5N1 VIRUSES

Early strains of HPAI H5N1 caused no or mild clinical symptoms in wild birds and waterfowl, such as ducks (10, 11, 48, 49). While wild birds and waterfowl species generally show resistance to HPAI H5N1, Galliformes (including domestic chickens, turkeys, and quails) exhibit particularly high susceptibility, demonstrating severe clinical disease with mortality rates frequently approaching 100% in outbreak settings (50–52). However, the clade 2.3.4.4b H5N1 has become well adapted to wild birds and waterfowl. Infections of wild birds and waterfowl with clade 2.3.4.4b H5N1 have been detected worldwide, with unprecedented case numbers and a high mortality rate (5, 14, 16, 53–55). According to the World Organization for Animal Health, clade 2.3.4.4b HPAI H5N1 caused over 11,400 outbreaks in poultry and wild birds across 84 countries between January 2022 and December 2023, resulting in significant economic losses in poultry populations and ecological damage in wild bird populations. This suggests that the current clade 2.3.4.4b H5N1 viruses exhibit an infection in a broad range of avian species.

Furthermore, clade 2.3.4.4b H5N1 viruses showed increased cases of infections in mammalian species. The virus has been isolated from wild red foxes, resulting in noticeable neurological symptoms after infection (7). In October 2022, an outbreak of clade 2.3.4.4b H5N1 occurred in a mink farm in Spain, leading to the culling of over 50,000 minks (9, 56, 57). The H5N1 viruses in minks showed efficient replication in minks and caused weight loss of 17.0%–37.9% (54). In November 2022, Peru reported large numbers of pelican and sea lion deaths caused by clade 2.3.4.4b HPAI H5N1 infection (15, 58). Additionally, New England also detected clade 2.3.4.4b HPAI H5N1 infection in seals (59). The recent detections of clade 2.3.4.4b HPAI H5N1 virus (genotype B3.13) in December 2024 in dairy farms across 15 states in the US raise concerns (60). Milking equipment is suspected as one of the possible transmission routes (61, 62). Alarmingly, several cases of H5N1 virus infection have been reported in individuals who had close contact with infected cattle, highlighting a serious public health threat (18, 63, 64). Altogether, this suggests that the current clade 2.3.4.4b H5N1 viruses show an increased host range in mammalian species, raising a heightened zoonotic risk.

**Fig 3:**
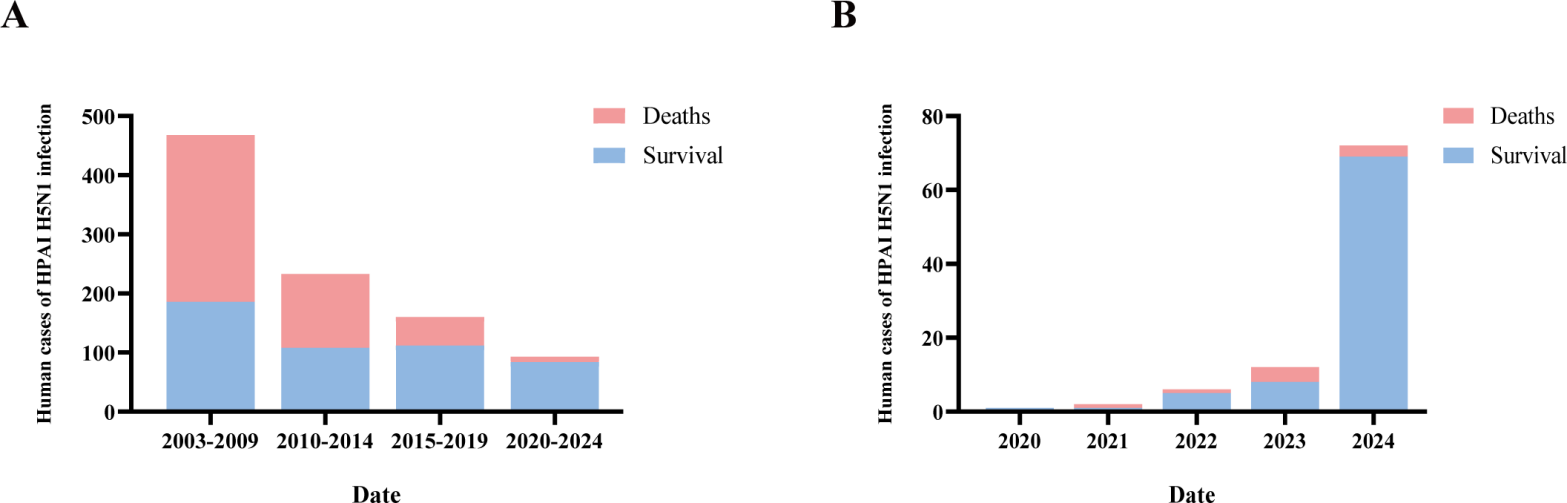
Cases of human infections with highly pathogenic H5N1 AIV. (**A**) From 2010 to 2024, statistics on human infections with HPAI H5N1 from 2010 to 2024, by every 5 years. (**B**) Statistics on human infections with HPAI H5N1 from 2020 to 2024, on an annual basis. All data are from the WHO and were plotted using GraphPad 8.3 software.

Although human infections of HPAI H5N1 viruses remain sporadic, there has been a recent increase. From January 2003 to December 2024, the World Health Organization (WHO) has recorded 954 confirmed human cases of HPAI H5N1 infection from 24 countries across Asia, Africa, Europe, and North America, resulting in 464 deaths (48.64% mortality rate; Fig. 3A). Notably, most of the cases occurred before 2016. Between 2020 and 2024, there were 93 new human HPAI H5N1 infections with an increasing trend year by year (Fig. 3B). These viruses were primarily derived from clade 2.3.4.4b (with infections mainly reported in the United States and the United Kingdom) and clade 2.3.2.1c (with infections mainly reported in Laos, Cambodia, and Vietnam; Table 1). Recent studies have identified multiple key amino acids in the HA of clade 2.3.4.4b H5N1 viruses that may play roles in mammalian adaptation (Table 2) (65, 66). Elucidating these molecular changes is crucial for understanding how the virus gained its increased ability to spread in animals and its greater likelihood of infecting humans. However, there are several key unknowns that impact our ability to anticipate the evolution of H5N1 viruses, particularly in relation to their adaptation to animal hosts and potential cross-species transmission. These include the mechanisms of adaptive mutations in viral surface proteins, the strategies of host immune evasion, the role of reassortment, and the influence of ecological factors on viral evolution. Addressing these uncertainties is essential for predicting future evolutionary trends and assessing the risks of cross-species transmission.

**TABLE 1:** Reported HPAI H5N1 human cases, January 2020 to May 2024^*a*^

Country	Report time	Disease severity and outcome	Virus clade	Number of cases per year
Laos	2020.10	Critical illness, survived	Clade 2.3.2.1c	1
India	2021.7	Critical illness, died	Clade 2.3.2.1a	2
United Kingdom	2021.12	Asymptomatic	Clade 2.3.4.4b	
United States	2022.4	Fatigue only, survived	Clade 2.3.4.4b	6
China	2022.9	Critical illness, died	Clade 2.3.4.4b	
Spain	2022.9	Asymptomatic	Clade 2.3.4.4b	
Spain	2022.10	Asymptomatic	Clade 2.3.4.4b	
Vietnam	2022.10	Critical illness, survived	Not reported	
Ecuador	2022.12	Critical illness, survived	Clade 2.3.4.4b	
China	2023.1	Hospitalized, outcome not reported	Clade 2.3.4.4b	12
Cambodia	2023.2	Critical illness, died	Clade 2.3.2.1c	
Cambodia	2023.2	Mild illness, survived	Clade 2.3.2.1c	
Chile	2023.3	Critical illness	Clade 2.3.4.4b	
United Kingdom	2023.5	Asymptomatic	Clade 2.3.4.4b	
United Kingdom	2023.5	Asymptomatic	Clade 2.3.4.4b	
United Kingdom	2023.7	Asymptomatic	Clade 2.3.4.4b	
United Kingdom	2023.7	Asymptomatic	Clade 2.3.4.4b	
Cambodia	2023.10	Critical illness, died	Clade 2.3.2.1c	
Cambodia	2023.10	Critical illness, died	Clade 2.3.2.1c	
Cambodia	2023.11	Critical illness, died	Clade 2.3.2.1c	
Cambodia	2023.11	Mild illness, survived	Clade 2.3.2.1c	
Cambodia	2024.1	Severe illness, survived	Clade 2.3.2.1c	7
Cambodia	2024.1	Severe illness, survived	Clade 2.3.2.1c	
Cambodia	2024.1	Critical illness, died	Clade 2.3.2.1c	
Cambodia	2024.2	Severe illness, survived	Not reported	
Cambodia	2024.2	Asymptomatic	Clade 2.3.2.1c	
Vietnam	2024.3	Critical illness, died	Clade 2.3.2.1c	
United States	2024.3	Mild illness, survived	Clade 2.3.4.4b	
Total				28

^*a*^ The above data are all sourced from WHO and the Centers for Disease Control and Prevention (https://www.cdc.gov/flu/avianflu/).

**TABLE 2:** Amino acid site mutations associated with the pathogenicity of H5N1^*a*^

Protein	Mutational site	Function
PB2	E627K^*b*^	Enhanced replication in mammalian cells (7, 67)
PB2	D701N^*b*^	Enhanced adaptability and transmission in mammalian (15, 59, 68)
PB2	S714R	Enhanced polymerase activity and replication efficiency in mammalian cells (69)
PB2	K702R	Enhanced adaptability and transmission in mammalian (70)
PB2	T271A^*b*^	Enhanced adaptability in mammalian receptors (71, 72)
PB2	Q591K^*b*^	Enhanced adaptability in human receptors (68)
PB1	G622D	Impeded the binding of PB1 protein with viral RNA, weakening the virulence of virus in mice (73)
PA	T165Y	Enhanced polymerase activity of the virus in mammalian cells as well as its virulence and pathogenicity in mice (74)
PA	M86I^*b*^	Enhanced adaptability of the virus in mammalian (15)
PA	S224P	Increased replication in duck embryo fibroblast cells (75)
PA	N383D	Increased polymerase activity in duck embryo cells and delayed the accumulation of PA and PB1 polymerase subunits in the nucleus of virus-infected cells (75)
PA	T515A	Enhanced adaptability in duck (76)
HA	S221P	Increased virus adaptability to bind with α2,6 sialic acid receptors (77–81)
HA	K216E	
HA	N158D^*b*^	
HA	N224K	
HA	T318I	
HA	H110Y	
HA	T160A	
HA	Q226L	
HA	G228S	
HA	S227N	
HA	D187G	
HA	E190G	
HA	Q196R	
HA	G186V	Enhanced binding capability of the H5 and H7 subtypes HA protein to human receptors, facilitating virus replication in both human and animal hosts (82)
HA	Q226L	
HA	G228S	
HA	T156A^*b*^	Enhanced virus adaptability to bind with α2,3 and α2,6 sialic acid receptors (83)
NA	49–68 deletions	Enhanced virulence in mice (84, 85)
NA	54–72 deletions	Enhanced virulence in mice but not chickens (86)
M1	D30N	Attenuated H5N1 influenza viruses in mice (87)
M1	A215T	
NS1	D26K^*b*^	Enhanced adaptability in mammalian (15)
NS1	R55K	Replicate with higher titers in human A549 cells and macrophages (88)
NS1	E66K	
NS1	F133C	

^*a*^ To enhance clarity, each row corresponds to a single amino acid substitution. The function and references listed are specific to the indicated mutation.

^*b*^ The amino acid positions that have been found to exist in the current clade 2.3.4.4b H5N1 viruses.

## “HOST-JUMP” ADAPTATIONS OF CLADE 2.3.4.4b HPAI H5N1 VIRUSES

For HPAIVs to achieve “host jump” from avian hosts to mammalian hosts, the virus often requires acquiring mammalian adaptations. HA glycoprotein has a critical role in virus entry into host cells and host transmission. AIVs often switch receptor binding specificity to overcome host barriers and change host tropism by acquiring mutations in HA. The binding of HA to sialic acid (SA) receptors is a prerequisite for AIV infection (89). The HA of human or mammalian viruses generally exhibits a binding preference toward α−2,6-linkage SA receptors, while the avian influenza viruses HA typically binds more efficiently to α−2,3-linkage SA receptors (90, 91). Recent risk assessment studies have indicated that the HA of clade 2.3.4.4b H5N1 viruses showed preferable binding to avian α−2,3-linkage SA receptors (92–94). However, most evidence suggests that key amino acid mutations in the HA of H5N1 viruses, such as N224K, Q226L, and G228S, can facilitate a switch in receptor-binding preference from avian to human and promote airborne transmission in mammals (Table 2) (81, 94–98). Although these mutations have not been commonly identified in clade 2.3.4.4b H5N1 viruses, their functional relevance remains important for risk assessment. Additionally, the T199L mutation in the HA of clade 2.3.4.4 H5N1 has been reported to enhance the breadth of α−2,3-linked SA receptor binding (99). This may be one of the key factors contributing to the adaptation of the 2.3.4.4b clade H5N1 virus to wild birds and waterfowl.

In addition to HA, polymerase genes (PB2, PB1, and PA) are also important for host adaptation and affecting host tropism (Table 2). The most studied mammalian adaptation, PB2 E627K, enhances virus polymerase activity and replication in mammalian cells and increases infectivity and transmissibility in mice and ferrets (81, 100–102). Several studies on current clade 2.3.4.4b H5N1 viruses have also identified the PB2 E627K mutation in cows, minks, and foxes (103–105). Moreover, PB2 D701N, Q591K, and T271A mutations have also been identified as potential mammalian adaptations in H5N1 viruses (106–108). Previous studies on current clade 2.3.4.4b H5N1 viruses have also demonstrated D701N, Q591K, M631L, and T271A mutations in sea lion, seal, mink, and human (9, 15, 59, 68). In the PA protein, mutations in M86I contributed to enhance the adaptability of mammals (74). Current studies on clade 2.3.4.4b H5N1 viruses have found the M86I mutation in sea lions and humans (15). However, research on the PB1 protein of clade 2.3.4.4b HPAI H5N1 has not been reported yet. Studies related to early HPAI H5N1 PB1 have mostly focused on restricting viral replication (73, 109). Although the main mechanisms by which clade 2.3.4.4 H5N1 virus’s cross species barriers to infect mammals are not yet fully understood, the increasing evidence of these mutations in mammals suggests that polymerase protein mutations may contribute to this adaptation, helping the virus cross species barriers and establish infections.

To prevent HPAI H5N1 from further adapting to wild birds and mammals, intensified monitoring of critical amino acid sites is essential. This will be crucial for controlling the spread of the virus and identifying early signs of adaptation. However, studying the synergistic effects of multiple mutations on transmissibility and virulence presents significant challenges. The interactions between multiple mutations are complex, and current experimental approaches are limited in their ability to fully capture these effects (94). Furthermore, functional studies of these interactions are difficult to perform, especially given the ethical and safety concerns surrounding experiments that involve gain-of-function (GOF) modifications. While GOF studies are valuable for understanding viral evolution, they also face technical limitations and pose potential risks that need to be carefully addressed (95). Therefore, it is important to acknowledge these limitations when considering future research on viral adaptation and pathogenicity.

## HOST FACTORS ASSOCIATED WITH HPAI H5N1 VIRUSES

HPAIV infection triggers a hyperinflammatory response characterized by cytokine storm, a phenomenon strongly associated with severe lung damage in avian and mammalian hosts (110, 111). Mechanistic studies have recently clarified this pathological process. Ruan et al. (112) demonstrated that H5N1 infection disrupted the alveolar epithelial barrier in mice by targeting intercellular junction proteins through a process called Itch-mediated proteasomal degradation. Similarly, Dai et al. (113) revealed that severe lung injury in chickens caused by clade 2.3.4.4b HPAI H5N1 arises from complex interactions between inflammatory macrophages, pro-inflammatory cytokines, and various cell types. Notably, experimental HPAI H5N1 infection in duck lung tissue exhibited a robust neutrophil response compared to infection with low pathogenic avian influenza virus(LPAIV) (114). These studies indicated differential cytokine profiles between host species, including notable responses in infected avian and mammalian hosts. Importantly, we have yet to completely understand the differences in the innate immune responses induced by 2.3.4.4b clade H5N1 virus infection among various hosts (especially waterfowl and mammals). Deeply understanding these immune response differences may help elucidate why clade 2.3.4.4b exhibits different pathogenicity across diverse hosts.

**Fig 4:**
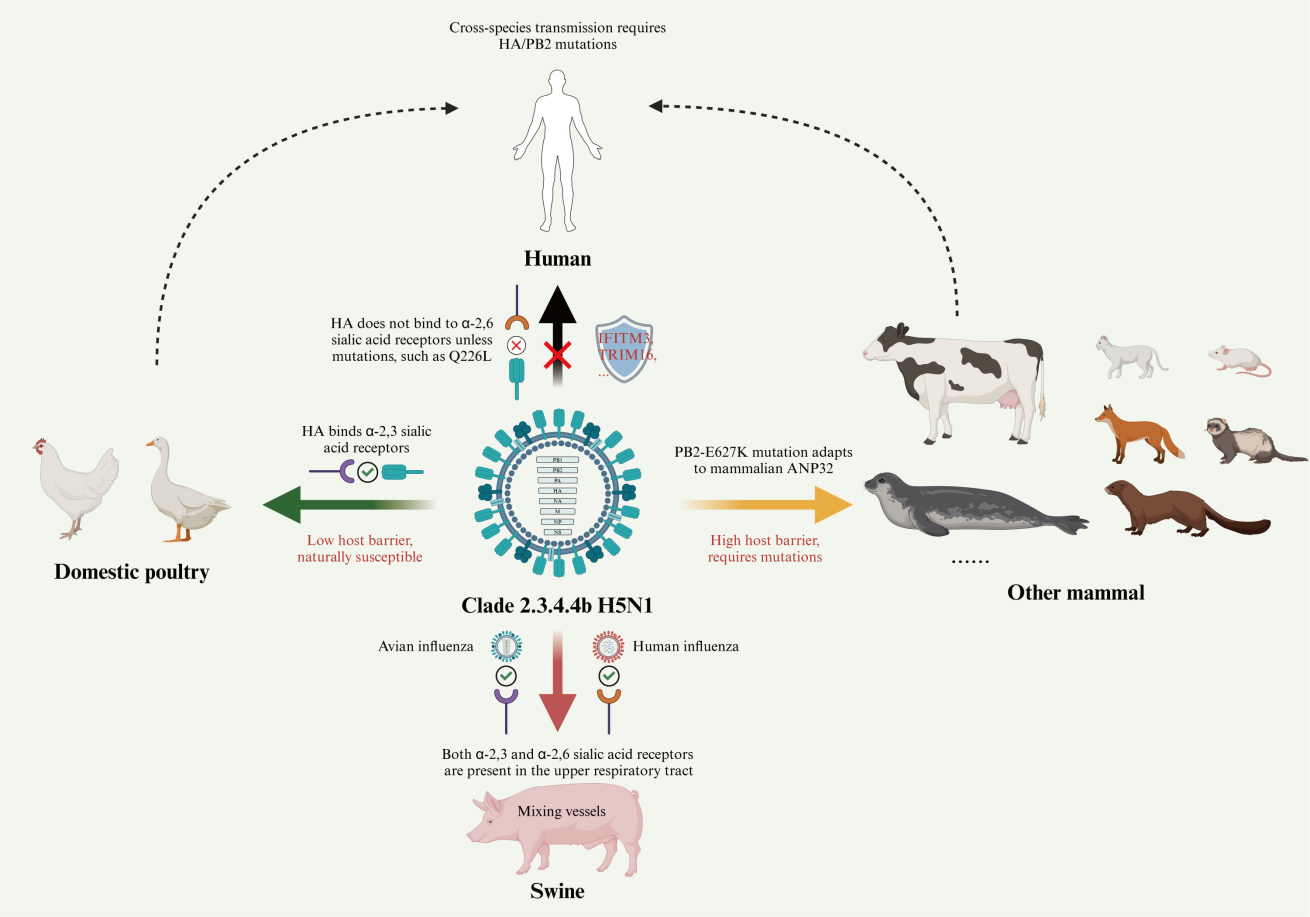
Host factors influencing the cross-species transmission of clade 2.3.4.4b H5N1 virus. Domestic poultry, such as chickens and waterfowl, is naturally susceptible to H5N1 due to low host barriers and the binding of HA to α−2,3 SA receptors. However, for human and most mammalian infections, H5N1 must undergo mutations to bind to α−2,6 SA receptors (e.g., HA-Q226L) and overcome high host barriers (e.g., PB2-E627K). Swine, as mixing vessels for avian and human influenza, possess both α−2,3 and α−2,6 SA receptors in the upper respiratory tract. Green arrows in the figure represent natural infection; black arrows indicate resistance to infection; yellow arrows indicate infection after mutation; and red arrows indicate the potential for new recombinant viruses to emerge after infection.

All viruses must obtain resources from host cells to replicate their genomes (Fig. 4). Studies have shown that the avian host factors acidic nuclear phosphoprotein 32 (ANP32) can specifically bind to the PB2 protein of H5N1, helping viral particles enter host cells and supporting viral replication (115–117). Although this specificity can create a barrier to the cross-species transmission of H5N1 (118), viral mutations may overcome this barrier, enabling the PB2 to bind to ANP32 from other species, such as the PB2-E627K mutation (94, 105, 119). Similarly, the host factor IGDCC4 enhances the internalization of H5N1 HA into host cells, contributing to increased viral replication and virulence (120). Additionally, annexin A2 has been shown to interact with the NS1 protein of H5N1, promoting viral protein synthesis and increasing progeny virus yield (121). Other host factors, such as the inhibitor of activated STAT2 (PIAS2), Staufen 2 (STAU2), and TGFβ-1 activated kinase 1 (TAK1), have also been identified as promoters of H5N1 replication (122–126). While the precise mechanisms remain to be fully clarified, sustained interactions between viral proteins and host factors may exert evolutionary pressure that promotes the emergence of adaptive mutations, thereby enhancing viral adaptation to specific hosts over time (127–129).

Conversely, the innate immune response provides early defense against H5N1 infection by initiating pattern recognition receptors that detect pathogen-associated molecular patterns within hours of infection. This leads to the activation of signaling cascades that transactivate pro-inflammatory cytokines to limit viral replication. Several host proteins, including tripartite motif-containing protein 16 (TRIM16) and TRIM21, have been shown to inhibit H5N1 replication by limiting viral protein synthesis (130, 131). Additionally, host proteins, such as interferon-induced transmembrane proteins 3, poly (ADP-ribose) polymerase family member 10, fibroblast growth factor receptor 1, and aryl hydrocarbon receptor nuclear translocator, are also associated with the suppression of viral replication (132–135). Additionally, some studies have shown that microRNAs like miR-21–3p and miR-324–5p can also inhibit H5N1 replication (136, 137). However, the inhibition generated by these interactions may also put pressure on H5N1, forcing the virus to continuously adapt and evolve in the host environment (138).

Although many host factors have been identified using previous HPAIVs, our understanding of host factors that influence the pathogenesis of current clade 2.3.4.4b H5N1 viruses remains limited. To address this critical gap in knowledge, we urgently need to elucidate why the virus can infect a broad range of hosts and identify the specific host factors that have evolved and their mechanisms of interaction with the virus.

## CONTROLLING HPAI CLADE 2.3.4.4b H5N1 VIRUSES

Controlling the spread of HPAI H5N1 in poultry is particularly challenging, requiring a combination of biosafety measures, surveillance, culling, and vaccination. While massive culling remains the most effective approach used in much of Europe and North America to control outbreaks (35), its long-term sustainability is questionable due to significant economic losses for poultry farmers, especially in endemic settings. Vaccination is a crucial strategy for controlling HPAI outbreaks, as it helps prevent the spread of the virus and reduces the impact on poultry populations. In China, a combination of culling and mass vaccination has been implemented as a primary control measure since 2004 (139). Similarly, countries such as Vietnam (2005), Indonesia (2004), Egypt (2006), and Mexico (2004) have also conducted large-scale poultry vaccination campaigns to control HPAI H5 subtype viruses (140–144). In 2023, the French government approved the use of H5N1 vaccines for ducks to further mitigate the spread of the virus.

However, the recent outbreak of HPAI H5N1 in dairy cattle has intensified concerns about the cross-species transmission of the virus. Studies have shown that dairy cows have a high concentration of avian α−2,3-linkage SA receptors in their mammary glands, making them vulnerable to H5N1 infection (145). Although pasteurization effectively eliminates the virus from milk, the quality of milk production is severely affected in infected cows, and the risk of virus transmission remains (146). To limit the spread of the virus, measures such as quarantine, enhanced surveillance, and early detection have been implemented. However, controlling H5N1 in dairy cattle still presents significant challenges, including the lack of effective vaccines, the high-risk environment for cross-species transmission, and difficulties in enforcing biosecurity on large-scale dairy farms. Therefore, developing effective vaccines, improving biosecurity measures, and implementing stricter animal management practices are critical in addressing this emerging threat.

Currently, vaccines with various formulations have been developed to combat the clade 2.3.4.4b H5N1 viruses. In China, commercially available inactivated whole-virus vaccines targeting the clade 2.3.4.4b H5 subtype, such as Re-14 and rGD59, are widely used for prevention (147). Notably, the vaccine used in China is updated whenever a distinct antigenic difference is observed between the vaccine strain and newly detected viruses. Moreover, recent developments include virus-like particle vaccines and nucleic acid vaccines targeting clade 2.3.4.4b H5N1 viruses, both of which have demonstrated efficient protective efficacy against H5N1 (148, 149). However, research on mRNA vaccines is still in its early stages, with studies mainly conducted in mammals (150–153). Although inactivated vaccines can effectively prevent HPAI H5N1 infections, their cross-protection is insufficient, and the rapid mutation of influenza viruses often leads to antigen-vaccine mismatches. Developing a vaccine that can induce both humoral and cellular immunity, while offering broad-spectrum protection, is the way forward. Therefore, mRNA vaccines, nanoparticle vaccines, and multi-epitope vaccines based on the conserved stalk region of HA may become key research focuses in the future (154, 155). Additionally, there has been growing interest in targeting NA as a potential vaccine target. NA is critical for viral release and spread, and vaccines targeting NA could provide additional protection, particularly in improving cross-protection and preventing resistance (156–158). Some studies have explored NA-based vaccine candidates, showing that immune responses directed against NA can help control H5N1 replication and transmission (159, 160). These findings highlight the potential of NA-based vaccines as an important addition to current influenza vaccine strategies, offering further opportunities for improving protection against H5N1 and other evolving strains.

Currently, the rapid evolution of HPAI H5N1 presents significant challenges to virus prevention and control. While vaccination can protect birds from virus infection, it may not prevent them from shedding the virus either oropharyngeal or cloacal (148, 161, 162). This often causes environmental contamination and may inadvertently select mutant viruses that can escape vaccine protection, leading to the emergence of new variants and vaccine failure. Moreover, one of the other difficulties is the repeated introduction of the virus from wild birds, which continuously threatens poultry populations (35, 163). Therefore, biosecurity measures, including proper surveillance, quarantine protocols, and disinfection practices, play a critical role in preventing the spread of HPAI H5N1 viruses. However, controlling outbreaks is complicated by factors such as the movement of animals, environmental contamination, and gaps in surveillance systems. Effective containment strategies must also address the challenges posed by both the high mutation rates of the virus and its ability to adapt in different hosts, making surveillance and rapid response crucial in limiting the impact of HPAI H5 outbreaks.

In summary, while widespread vaccination has the potential to accelerate the evolution of H5N1 viruses and drive the emergence of vaccine-escape variants, culling—as the primary control strategy in many Western countries—imposes substantial economic burdens. Thus, a comprehensive understanding of the interplay between vaccination and viral evolution is poised to become a critical focus of future research efforts.

## CONCLUDING REMARKS

Clade 2.3.4.4b HPAI H5N1 viruses have caused unprecedented outbreaks worldwide since October 2020. The virus has been widely identified in wild birds, domestic poultry, and many mammalian species. We have outlined our understanding of the current clade 2.3.4.4b H5N1 virus evolution, host range, molecular marker, host factors, and virus control by vaccination. However, it remains unclear why the virus is so widespread and infectious in various hosts and regions, and also, the potential risks of the rapidly evolved virus to animals and humans remain unknown. Therefore, surveillance and risk assessment remain crucial for understanding the potential threats posed by the viruses, and vaccine development and other controlling strategies are also urgently needed to mitigate the virus impact on public health and agricultural sectors.

## SUPPLEMENTAL MATERIAL

Data S1 jvi.00424-25-s0001.xlsx Sequence information of the phylogenetic tree for avian influenza A (H5N1) virus from GISAID EpiFlu database (1997-2024).

ASM does not own the copyrights to Supplemental Material that may be linked to, or accessed through, an article. The authors have granted ASM a non-exclusive, world-wide license to publish the Supplemental Material files. Please contact the corresponding author directly for reuse.
